# Characterization and function of the human macrophage dopaminergic system: implications for CNS disease and drug abuse

**DOI:** 10.1186/1742-2094-9-203

**Published:** 2012-08-18

**Authors:** Peter J Gaskill, Loreto Carvallo, Eliseo A Eugenin, Joan W Berman

**Affiliations:** 1 Department of Pathology, Albert Einstein College of Medicine, Bronx, NY, USA; 2 Public Department of Microbiology and Molecular Genetics, Health Research Institute, UMDNJ, Newark, NJ, USA; 3 Department of Microbiology and Immunology, Albert Einstein College of Medicine, Bronx, NY, USA

**Keywords:** Monocyte derived macrophage, Dopamine, Drug abuse, Cytokine, Neuroinflammation dopamine receptor, Dopamine transporter, Tyrosine hydroxylase, Aromatic amino acid decarboxylase

## Abstract

**Background:**

Perivascular macrophages and microglia are critical to CNS function. Drugs of abuse increase extracellular dopamine in the CNS, exposing these cells to elevated levels of dopamine. In rodent macrophages and human T-cells, dopamine was shown to modulate cellular functions through activation of dopamine receptors and other dopaminergic proteins. The expression of these proteins and the effects of dopamine on human macrophage functions had not been studied.

**Methods:**

To study dopaminergic gene expression, qRT-PCR was performed on mRNA from primary human monocyte derived macrophages (MDM). Expression and localization of dopaminergic proteins was examined by immunoblotting isolated plasma membrane, total membrane and cytosolic proteins from MDM. To characterize dopamine-mediated changes in cytokine production in basal and inflammatory conditions, macrophages were treated with different concentrations of dopamine in the presence or absence of LPS and cytokine production was assayed by ELISA. Statistical significance was determined using two-tailed Students’ T-tests or Wilcoxen Signed Rank tests.

**Results:**

These data show that MDM express mRNA for all five subtypes of dopamine receptors, and that dopamine receptors 3 and 4 are expressed on the plasma membrane. MDM also express mRNA for the dopamine transporter (DAT), vesicular monoamine transporter 2 (VMAT2), tyrosine hydroxylase (TH) and aromatic amino acid decarboxylase (AADC). DAT is expressed on the plasma membrane, VMAT2 on cellular membranes and TH and AADC are in the cytosol. Dopamine also alters macrophage cytokine production in both untreated and LPS-treated cells. Untreated macrophages show dopamine mediated increases IL-6 and CCL2. Macrophages treated with LPS show increased IL-6, CCL2, CXCL8 and IL-10 and decreased TNF-α.

**Conclusions:**

Monocyte derived macrophages express dopamine receptors and other dopaminergic proteins through which dopamine may modulate macrophage functions. Thus, increased CNS dopamine levels due to drug abuse may exacerbate the development of neurological diseases including Alzheimer’s disease and HIV associated neurological disorders.

## Background

In individuals who abuse drugs such as cocaine and methamphetamine [1,2], extracellular dopamine in different regions of the brain is increased, exposing perivascular macrophages and microglia to elevated concentrations of dopamine [3-5]. Central nervous system (CNS) dopamine is also increased by therapeutic drugs including antidepressants [6], L-DOPA [7,8], methylphenidate [9], cholinergic agents for treatment of Alzheimer’s disease [10], as well as by stress [11], depression [12,13] and neuropathologies such as HIV-associated neurological disorders (HAND) [14] and schizophrenia [15,16]. Little is known about the effects of dopamine on human macrophages, although studies in rodent macrophages and microglia, as well as in human T-cells indicate that exposure to high concentrations of dopamine significantly impacts the function of macrophages and microglia [17-24]. Perivascular macrophages and microglia are central to CNS function [25], and dysfunction in these cell types is associated with the development of many neurological disorders [26,27]. Therefore, we characterized the macrophage dopaminergic system and examined the effects of dopamine on macrophages to define mechanisms by which increases in CNS dopamine may contribute to the development of neuropathology.

Dopamine is synthesized by conversion of tyrosine to L-DOPA by the enzyme tyrosine hydroxylase (TH), and L-DOPA is then converted to dopamine by aromatic amino acid decarboxylase (AADC) [28,29]*.* Dopamine acts through activation of dopamine receptors (DR), which are divided into two sub-classes, D1-like dopamine receptors, D1R and D5R, and D2-like dopamine receptors, D2R, D3R and D4R [30]. Dopamine receptor activation depends on CNS dopamine concentrations, which are regulated by dopamine transporter (DAT)-mediated re-uptake, metabolic breakdown by monoamine oxidases and catechol-O-methyl transferase, and diffusion into extracellular fluid [31-34]. Recaptured or newly synthesized dopamine is transported from the cytoplasm into secretory granules by vesicular monoamine transporters (VMAT), where it is stored until released [35]. Thus, the dopaminergic proteins DAT, VMAT, TH and AADC act together with DR to regulate the effects of dopamine.

Dopamine receptors are expressed on human T-cells, neutrophils, monocytes and B-cells [36]. T-cells also express TH and DAT, and take up, store, and synthesize dopamine as part of their regulatory processes [17,37-39]. Dopamine mediates proliferation, quiescence, chemotaxis and cytokine production in different subtypes of human T-cells [20,21,40-43] and also modulates neutrophil migration and apoptosis [44,45]. Expression of DAT, VMAT2 and AADC were detected in human myeloid cells as well as the promyelocytic U937 cell line [46,47]. Tyrosine hydroxylase and VMAT2 have been found in CD163+ human macrophages from arthritic synovial tissue but not in CD163+ cells from non-arthritic controls [48]. We previously demonstrated that primary human monocyte-derived macrophages (MDM) express D2-like DR on the cell surface and activation of these receptors increases HIV replication [49]. The effects of dopamine on human macrophages are not well characterized.

To examine the response of human macrophages to the increased dopamine levels induced by drug abuse, we characterized gene and protein expression of DR, DAT, VMAT2, TH and AADC in MDM. We also studied dopaminergic effects on cytokine production in both basal and inflammatory conditions by using untreated and LPS-treated macrophages. Macrophages have been shown to express mRNA for all subtypes of DR and have D3R and D4R on the plasma membrane. Macrophages also expressed mRNA and protein for DAT, VMAT2, TH and AADC, with DAT on the plasma membrane and VMAT2 in cellular membranes. Our data demonstrated that dopamine treatment significantly increased IL-6 and CCL2 in both untreated and lipopolysaccharide (LPS)-treated MDM, increased CXCL8 and IL-10 and decreased TNF-α in LPS-treated MDM. These data indicate that dopamine is an important mediator of both macrophage homeostasis and of the response of macrophages to injury and infection, and that drug-induced changes in CNS dopamine may alter the development of neurological disease.

## Methods

### Reagents

RPMI-1640 medium and penicillin/streptomycin (P/S) were from Invitrogen (Carlsbad, CA, USA). LPS from E.Coli 055:B5, hydroxyethyl piperazineethanesulfonic acid (HEPES), fish gelatin, β-mercaptoethanol, IgG-free bovine serum albumin (BSA), horse serum, Tween 20 and dopamine hydrochloride (DA) were obtained from Sigma-Aldritch (St. Louis, MO, USA). Dopamine was resuspended at 20 mM in dH2O, aliquoted and stored at −80°C for up to 2 months before use. Fetal calf serum (FCS) and human AB serum were from Lonza (Basel, Switzerland). Macrophage colony stimulating factor (M-CSF) was from Peprotech (Rocky Hill, NJ, USA). Antibodies used were rabbit polyclonal anti-DR3 (1:250), rabbit polyclonal anti-DR4 (1:250), rabbit polyclonal anti-VMAT2 (1:250) and rabbit polyclonal anti-AADC (1:200) (Millipore, Billerica, MA, USA), rabbit polyclonal anti-TH (1:500) (Cell Signaling Technology, Danvers, MA, USA), rabbit polyclonal anti-DAT (1:250 Western blot, 1:50 Immunofluoresence, Sigma), anti α-tubulin (1:10,000), fluorescein isothiocyanate (FITC)-conjugated anti-rabbit secondary antibodies (Sigma), anti-rabbit IgG and anti-rabbit Na^+^/K^+^ ATPase (1:200, Santa Cruz Biotechnologies, Santa Cruz, CA, USA). Other reagents were anti-fade with 4',6-diamidino-2-phenylindole (DAPI), TRIzol reagent and phalloidin conjugated to Texas red, obtained from Molecular Probes (Invitrogen).

### Generation of primary human macrophages

Human peripheral blood mononuclear cells (PBMC) were separated from blood obtained from de-identified healthy donors (New York Blood Center, Long Island City, New York, USA) by Ficoll-Paque (GE Healthcare, Piscataway, NJ, USA) gradient centrifugation. After isolation of PBMC, MDM were obtained either by adherence over 3 days or by using CD14 magnetic beads (EasySep Human CD14 Positive Selection kit), according to the manufacturer’s protocol (Stem Cell Technologies, Vancouver, Canada). MDM were cultured in RPMI-1640 with 10% FCS, 5% human AB serum, 10 mM HEPES, 1% P/S, and M-CSF (10 ng/mL) for 3 days, washed twice with fresh media, and cultured for another 3 days in fresh media containing M-CSF. After 6 days in culture cells were washed again and considered to be mature MDM.

### Quantitative RT-PCR

Total RNA was extracted from MDM using TRIzol (Invitrogen). Purity and concentration were determined using a Nanodrop spectrophotometer (Nanodrop technologies, Wilmington, DE, USA). On the same day of the extraction, 2 μg of RNA were used to synthesize cDNA from each donor using the iScript cDNA synthesis kit (Bio-Rad Laboratories Inc., Hercules, CA, USA), generating random cDNA fragments using both oligo-dT and random primers. FirstChoice Human Brain Total RNA was used as a positive control (Ambion, Austin, TX, USA) and samples containing no cDNA served as the negative controls. Target mRNA was quantified with the Absolute Blue QPCR SYBR low ROX Mix (Thermo Scientific). Single-product amplification was confirmed by melting-curve analysis. Reactions in which the negative control showed amplification curves that were detected before 37 cycles were not used. Specific dopaminergic and housekeeping genes were amplified from cDNA by quantitative PCR (qPCR) from 1 μL of cDNA reaction using gene-specific primers designed using Primer3 [50] and synthesized by Fisher Custom Oligonucleotides service (Table 1). The specificity of each primer for the gene of interest and all common splice variants was confirmed using a nucleotide BLAST search [51].

**Table 1:** Primers used for qRT-PCR

**Primer**	**Forward primer sequence (5′ - 3′)**	**Reverse primer sequence (5′-3′)**
β-actin	CTCTTCCAGCCTTCCTTCCT	AGCACTGTGTTGGCGTACAG
DRD1	AGGGGAATTTGCAGTTCTGT	AAAAGATGGAGAGGGCCAAT
DRD2	GCAGACCACCACCAACTACC	CCACTCACCTACCACCTCCA
DRD3	CACTGTCTGCTCCATCTCCA	GAGGATCCTTTTCCGTCTCC
DRD4	CCTTCTTCGTGGTGCACAT	AACTCGGCGTTGAAGACAGT
DRD5	GCCTACCAGAGATGGACCAA	AAAAGGGAGGGGAGAGCATA
TH	GTGAGGTTGTGCTGCCTGT	CTTTTATTGTGACGGTGATTGG
AADC	CGAGCAGAGAGGGAGTAGGA	CCACAGACAGCTGAGTTCCA
VMAT2	TTGAGGGTTTCTGGTTCTCC	ATACCTTTGCCAGGCCTTCT
DAT	CGAGCCTGCTTGCTGATATT	ATGGCATCCACTTTCCTGTC

### Immunofluoresence and confocal microscopy

MDM cultured on 35-mm Mat Tek dishes (Mat Tek, Ashland, MA, USA) were fixed and permeabilized in 70% ethanol for 30 minutes at 4°C. Cells were washed in PBS, incubated in block solution (0.5 M EDTA, 1% human AB serum, 2% fish gelatin, 1% Ig-free BSA, 1% horse serum in H2O) for 30 minutes at room temperature (RT) and then in primary antibodies (anti-DAT or isotype-matched controls, all 1:50) overnight at 4°C. Cells were washed with PBS and incubated for 1 hr at RT with the appropriate secondary antibody conjugated to FITC (1:250, Sigma) as well as phalloidin-conjugated Texas Red (1:50, Invitrogen). Cover slips were mounted on Mat Tek dishes using Prolong Gold Antifade reagent with DAPI (Invitrogen). MDM were examined by confocal microscopy using a Leica microscope (Wetzlar, Germany).

### Subcellular fractionation

Isolation of MDM plasma membrane, total membrane and cytosolic fractions was performed using a polyethylene glycol and dextran T-500-based centrifugal gradient kit, according to the manufacturer's protocol (BioVision Membrane Protein Extraction kit, Biovision, Mountain View, CA, USA). For each lysate, 20 to 40 × 10^6^ MDM cultured at 10 × 10^6^ MDM per dish were used to generate either a single plasma membrane or total membrane and cytoplasmic lysate.

### Immunoblotting

Human MDM were lysed with mammalian protein extraction reagent (M-PER, Pierce, Rockford, IL, USA) with added protease (Halt Protease inhibitor cocktail, Pierce) and phosphatase inhibitors (Halt Phosphatase Inhibitor Cocktail, Pierce). For detection of TH and AADC, lysates were sonicated, centrifuged at 14,000 × g, and stored at −80°C until used. Loading buffer containing β-mercaptoethanol was added to lysates that were then heated to 100°C for 5 minutes. Western blot analyses were performed using 4 to 12% Nupage polyacrylamide gels (Invitrogen). Proteins were transferred to polyvinylidene fluoride (PVDF) membranes (Invitrogen) and blots were blocked in Tris-buffered saline (TBS-T) (1×TBS with 1.2% Tween 20) containing 3% BSA/5% non-fat powdered milk. Signal was detected using Supersignal WestPico Chemiluminescent substrate for TH or Supersignal WestFemto Chemiluminescent substrate for AADC (Pierce). After probing for TH or AADC, blots were stripped using Restore PLUS Western Stripping Buffer (Pierce) and probed for α-tubulin as a loading control.

Plasma membrane and cytosolic lysates from the same donor were probed for either D3R, D4R or DAT, while total membrane and cytosolic fractions from the same donor were probed for VMAT2. Denaturing lysates by heating altered the mobility of the DR, DAT and VMAT2. Therefore, in place of heating, lysates were incubated in loading buffer containing β-mercaptoethanol for 45 minutes at RT. Western blots were run as described above, and after probing for DR or DAT, blots were stripped and reprobed for Na^+^/K^+^ ATPase and α-tubulin to confirm the specificity of the membrane isolations.

### Quantification of cytokine or chemokine secretion

MDM cultured at 5 × 10^5^ per well in 48-well plates (BD-Falcon) were incubated for 24 hrs with different concentrations of dopamine (20 nM, 200 nM, 2 μM or 20 μM) in the presence or absence of LPS (1 ng/mL). LPS treatment was used to model inflammation. Controls were treated with 1 ng/mL LPS without dopamine, or were untreated. After 24 hours, supernatants were collected, aliquoted and stored at −80°C. Supernatants were analyzed for IL-6, CCL2, TNF-α, CXCL8 and IL-10 using sandwich capture ELISAs was performed according to the manufacturer's protocol (Duoset ELISA Development systems, R&D systems, Minneapolis, MN, USA). The limits of detection for ELISAs were IL-6, 23.44 pg/mL, CCL2, 31.25 pg/mL, TNF-α, 15.625 pg/mL, CXCL-8, 31.25 pg/mL and IL-10, 62.5 pg/mL.

### Statistical analyses

Statistical analyses were performed using Prism 5.0 (Graphpad, La Jolla, CA, USA), using either a Wilcoxen Signed Rank Test (for experiments with N ≥10) or a two-tailed Student’s T-test (for all other experiments). P <0.05 was considered significant. To evaluate dopamine mediated changes in each cytokine, the fold-changes in the amount of each factor upon dopamine treatment from all donors were pooled and compared to the combined mean fold-change in the control condition (either untreated MDM or MDM treated with 1 ng/mL LPS), which was set to one.

## Results

### Primary human macrophages express dopamine receptors

**Figure 1:**
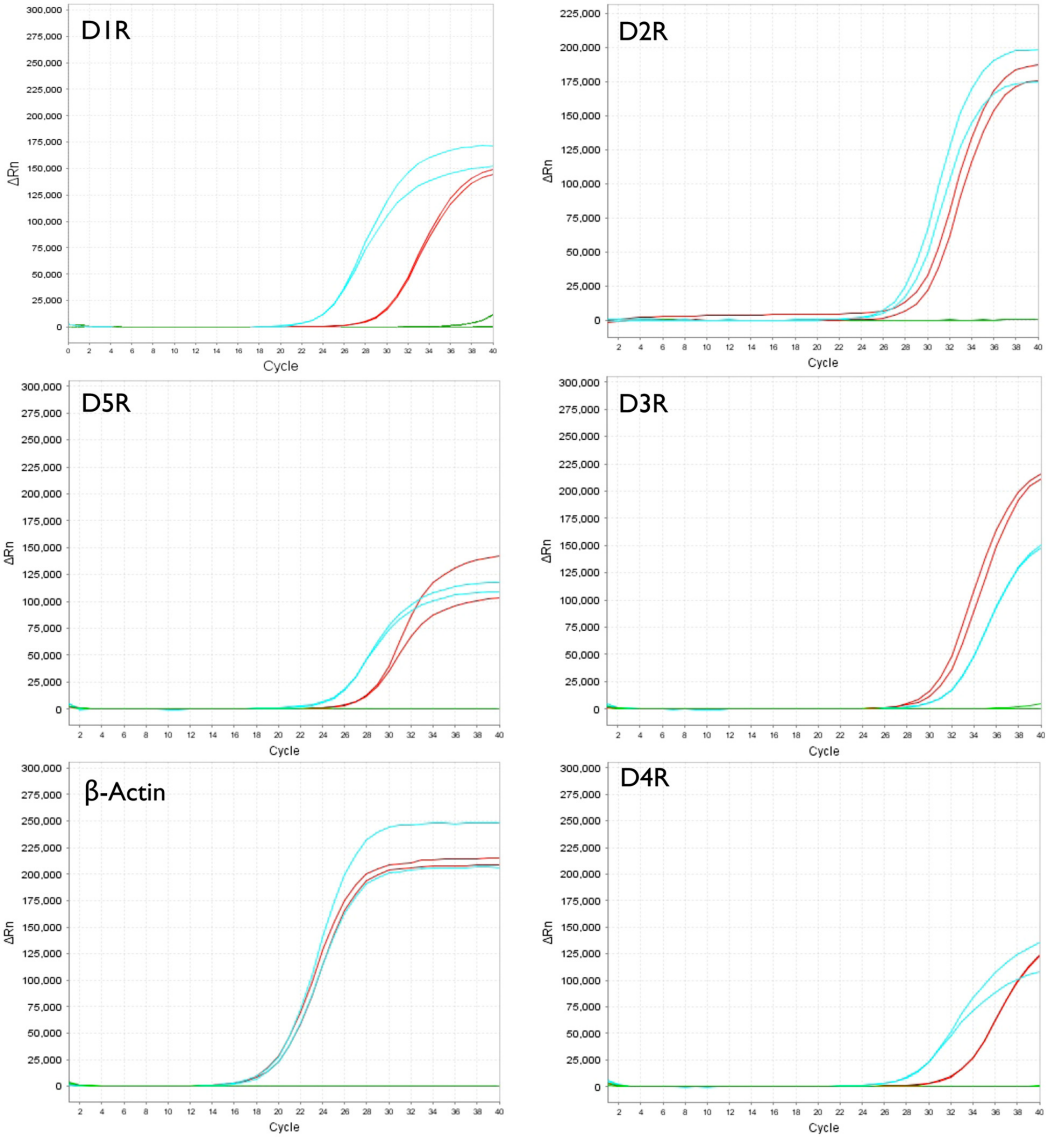
**Human macrophages express mRNA for all dopamine receptors (DRs).** Quantitative RT-PCR (1A) detected mRNA for D1R, D2R, D3R, D4R and D5R in monocyte derived macrophages (MMD) isolated from 4 different donors. Representative amplification curves from a single donor are shown for each DR (red curves) and the housekeeping gene β-actin (red curves). Amplification curves for the positive control (Total Human brain RNA, blue curves) and the negative control (cDNA negative samples, *green* curves) are also shown.

We previously showed that dopamine treatment of human macrophages significantly increases HIV replication [49], and others demonstrated that dopamine alters cytokine secretion and other functions in rodent macrophages and microglia as well as in human T-cells. Dopamine mediates its effects principally through activation of dopamine receptors. Therefore we examined the expression and localization of DR in human macrophages. Our previous data demonstrated D1R and D2R on the surface of MDM [49]. To expand upon our original studies, we examined human macrophage mRNA for D3R, D4R and D5R as well as for D1R and D2R. We also examined the expression and localization of D3R and D4R on MDM. Protein expression and localization of D5R was not examined because of the lack of available antibodies that distinguish specifically between D1R and D5R. The mRNA from MDM isolated from 4 different donors was analyzed by qRT-PCR for D1R, D2R, D3R, D4R and D5R, as well as for the housekeeping gene β-actin as a control for proper PCR amplification. Representative plots showing the mRNA amplification from a single donor for each DR or for β-actin (red curves) are shown in Figure 1. Total Human brain RNA was used as a positive control in each amplification reaction (blue curves) and samples containing no cDNA served as the negative control (green curves) in each reaction. Among the four donors examined, the ΔC_T_ relative to β-actin for D1R was 12 +/− 2.4 (mean +/− SD), for D2R was 16.6 +/− 4.2, for D3R was 12.6 +/− 3.7, for D4R was 14.7 +/− 3.7 and for D5R was 9.6 +/− 3.5. The negative control did not show amplification curves before 37 cycles in any amplification reaction.

**Figure 2:**
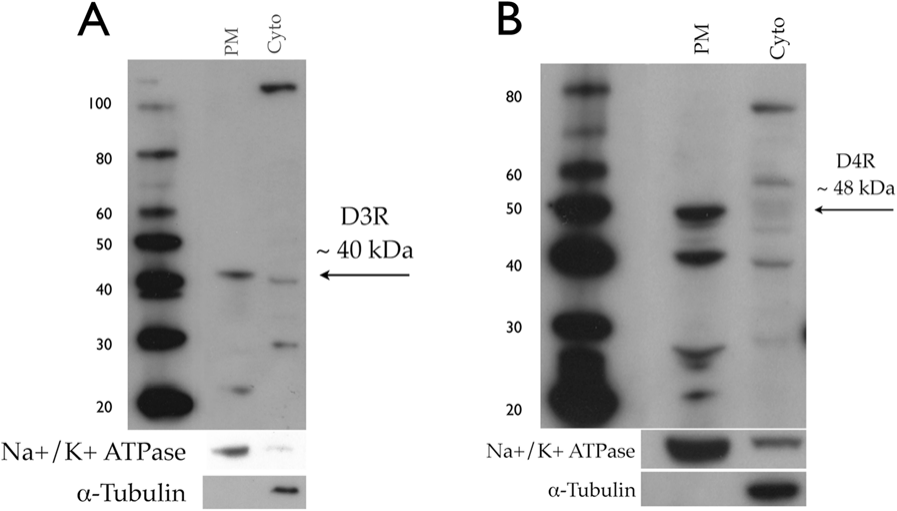
**Human macrophages express dopamine receptors (DRs) 3 and 4 on the plasma membrane. ** Western blot analysis of isolated plasma membrane (PM) and cytoplasmic (Cyto) proteins from monocyte-derived macrophages (MDM) demonstrated that D3R (*2 *B, black arrow, ~ 40 kDA) and D4R (*2 *C, black arrow,~ 48 kDA) were expressed in the PM. Specificity of the membrane protein isolation was confirmed by enrichment of Na^+^/K^+^ ATPase and lack of α-tubulin in plasma membrane fractions (B, C).

To demonstrate that D3R and D4R are expressed on the plasma membrane of MDM, plasma membrane (PM) proteins were examined by western blotting. Representative Western blots show both D3R (Figure 2A, black arrow, 40 kDA) and D4R (Figure 2B, black arrow, 48 kDA) in the PM fraction. The western blots were stripped and probed for the plasma membrane protein Na^+^/K^+^ ATPase, which was enriched in the PM fraction, and the cytosolic protein α-tubulin, which was not found in the PM fraction, confirming the localization of the DR in the PM. These data demonstrate that human MDM express mRNA for all types of DR, and that D3R and D4R are present in the plasma membrane.

### Primary human macrophages express tyrosine hydroxylase and aromatic amino acid decarboxylase

**Figure 3:**
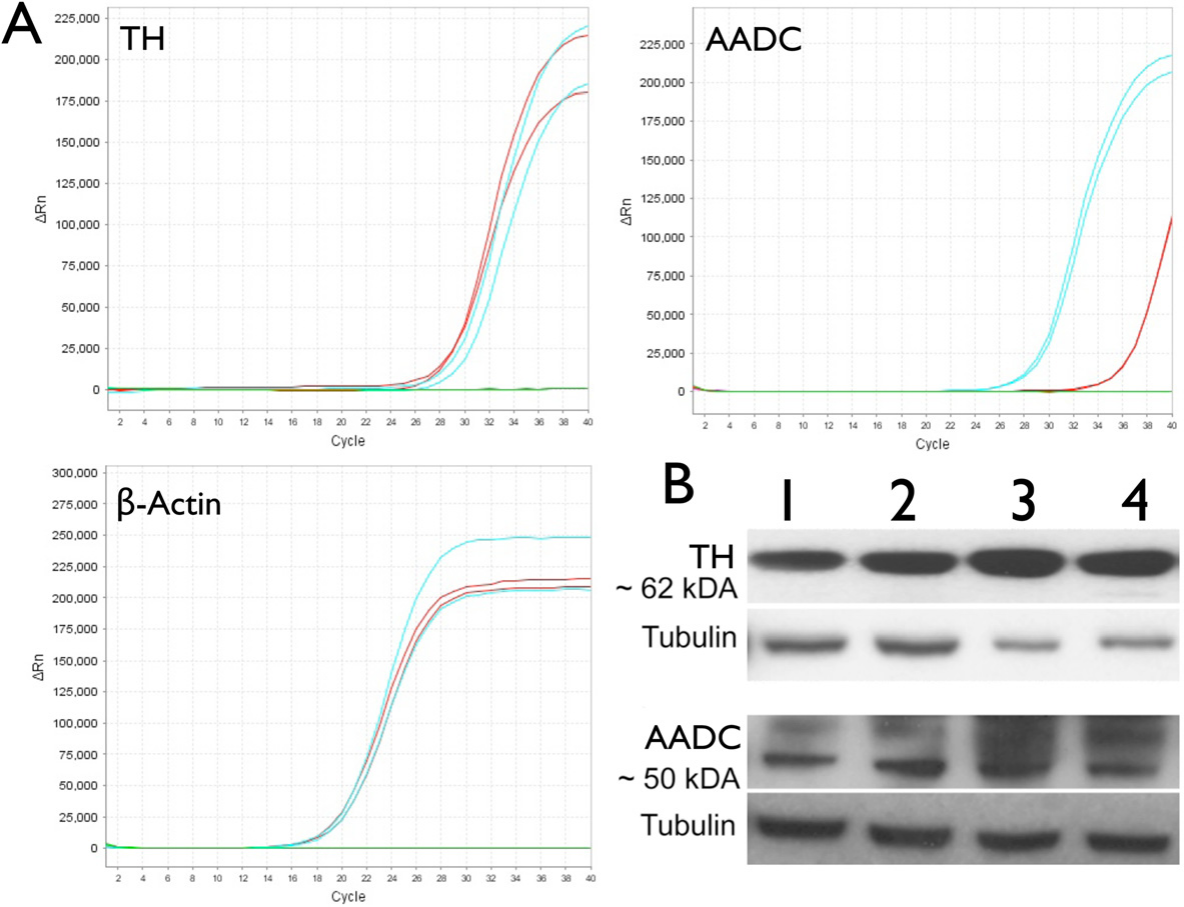
**Human macrophages express tyrosine hydroxylase and aromatic amino acid decarboxylase.** Quantitative RT-PCR (**A**) detected mRNA for tyrosine hydroxylase (TH) and aromatic amino acid decarboxylase (AADC) in monocyte derived macrophages (MDM) isolated from four different donors. Representative amplification curves from a single donor (A) are shown for TH, AADC (red curves) and the housekeeping gene β-actin (red curves). Amplification curves for the positive control (Total Human brain RNA, blue curves) and the negative control (cDNA negative samples, green curves) are also shown (A). Western blot analysis of MDM lysate from donors 1, 2, 3 and 4) (**B**) showed expression of TH (approximately 62 kDA) and AADC (approximately 50 kDA) in all donors examined.

To determine whether primary human macrophages express TH and AADC, and therefore have the capacity to synthesize dopamine, mRNA and protein for both were examined in human MDM from 4 different donors. Quantitative RT-PCR was used to amplify mRNA for TH and AADC, and representative amplification curves for TH, AADC and β-actin (red curves) from a single donor are shown in Figure 3A. Total human brain RNA and cDNA negative samples used as the positive (blue curves) and negative controls (green curves) are also shown in each plot. In mRNA derived from four donors, the ΔC_T_ relative to β-actin was 14 +/− 4.6 for TH and 21.1 +/− 3.8 for AADC. The negative control did not show amplification curves detected before 37 cycles in any amplification reaction. Western blot analyses demonstrated both TH and AADC in MDM whole cell lysate derived from four different donors (Figure 3B, donors 1, 2, 3, 4 TH approximately 62 kDA, AADC approximately 50 kDA). Blots were probed for α-tubulin as a loading control. These data demonstrate that primary human macrophages express the proteins required to synthesize dopamine.

### Primary human macrophages express dopamine transporter and vesicular monoamine transporter 2

**Figure 4:**
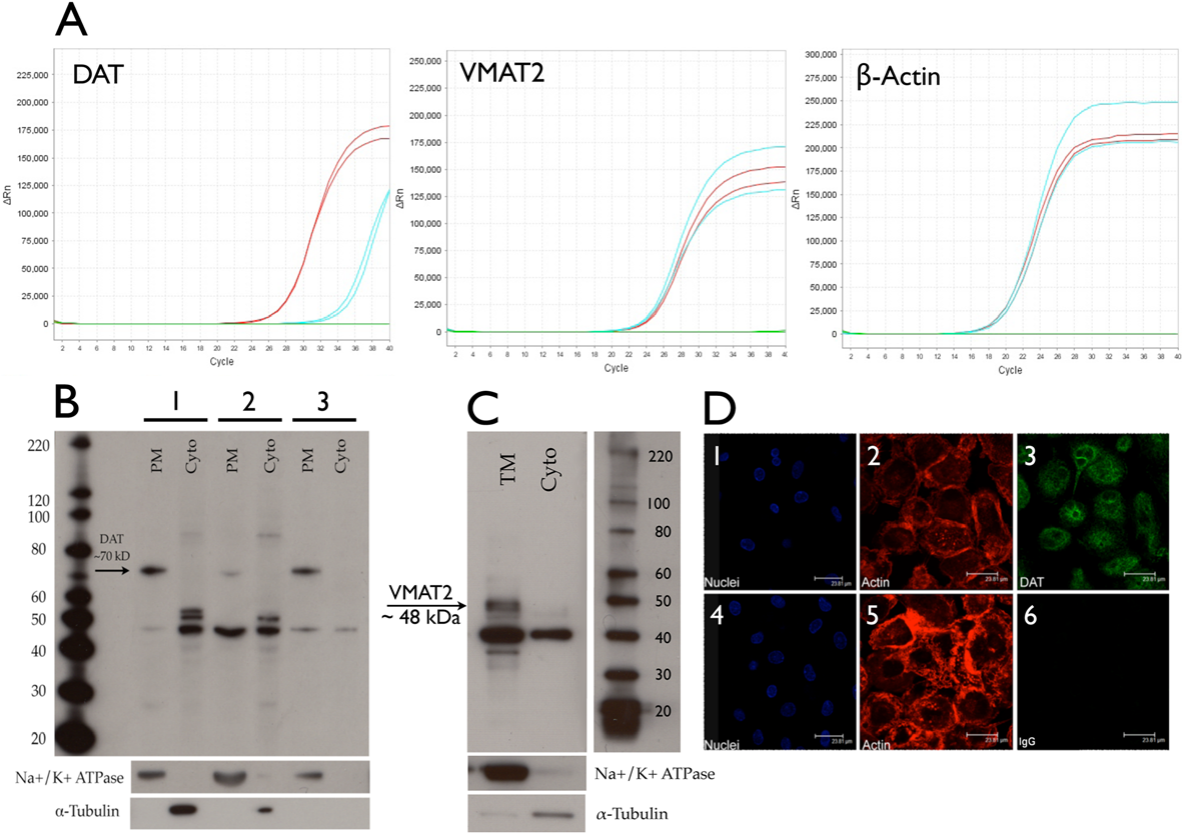
**Macrophages express the dopamine transporter and vesicular monoamine transporter 2.** Quantitative RT-PCR (**A**) detected mRNA for the dopamine transporter (DAT) and vesicular monoamine transporter 2 (VMAT2) in monocyte-derived macrophages (MDM) isolated from four different donors. Representative amplification curves from a single donor (A) are shown for DAT, VMAT2 (red curves) and the housekeeping gene β-actin (red curves). Amplification curves for the positive control (Total Human brain RNA, blue curves) and the negative control (cDNA negative samples, green curves) are also shown (A). Western blot analysis of isolated MDM plasma membrane (PM) and cytoplasmic (Cyto) proteins from three different donors demonstrated that DAT (**B**, black arrow, approximately 70 kDA) was expressed in the plasma membrane of MDM. Western blot analysis of isolated MDM total membrane (TM) and cytoplasmic (Cyto) proteins demonstrates expression of VMAT2 (4**C**, black arrow, approximately 48 kDA) in MDM membranes. Specificity of plasma and total membrane isolation was confirmed by enrichment of Na^+^/K^+^ ATPase and lack of α-tubulin reactivity in membrane fractions (B, C). Expression of DAT is also shown in MDM stained for DAT (4D-3, FITC) or the isotype matched negative control antibody (3D-6, FITC). Cell nuclei were stained with DAPI (4**D**-1, -4, blue), actin was stained with Phalloidin-Texas Red (4D-2,-5, red), and the DAT or the isotype control were stained with FITC (4D-3, 6, green). Scale bars represent 23.81 μM.

To determine whether MDM have the capacity to take up dopamine from the extracellular space to the cytoplasm and transport it into storage vesicles, we analyzed expression of DAT and VMAT2 mRNA and protein from MDM derived from three different donors. Dopamine transporter was also examined by immunofluoresence. Using qRT-PCR, DAT and VMAT2 mRNA were detected in all donors examined, and representative amplification curves of these genes (red curves) and the housekeeping gene β-actin (red curve) are shown in Figure 4A. Total Human brain RNA (blue curves) and cDNA–negative (green curves) samples were used as the positive and negative controls, respectively. The ΔC_T_ relative to β-actin was 11.2 +/− 5.7 for DAT and 8.5 +/− 3 for VMAT2. The negative control did not show amplification detected before 37 cycles in any reaction.

Western blot analysis of plasma membrane proteins isolated from three different donors shows DAT (Figure 4B, donors 1, 2, 3, black arrow, approximately 70 kDA) and VMAT2 (Figure 4C, black arrow, approximately 48 kDA) in the membrane fraction (PM, plasma membrane or TM, total membrane). We probed for VMAT2 in the total membrane fraction rather than the PM fraction because VMAT2 is found in cytoplasmic vesicles (ref for VMAT2). Neither protein was found in the cytosolic fraction (Cyto). Purity of the membrane isolation was confirmed by enrichment of Na^+^/K^+^ ATPase and the absence of α-tubulin in the membrane fractions. Dopamine transporter expression on MDM was also demonstrated by immunofluorescence. Figure 4D, panels 1–3, show DAT on the cell surface and in the cytoplasm, with cell nuclei labeled blue (4D-1 and 4D-4, DAPI), actin red (4D-2 and 4D-5, Texas Red) and DAT labeled green (4D-3, FITC). The isotype-matched control antibody had minimal to no reactivity (4D-6, FITC). These data demonstrate that primary human macrophages express DAT and VMAT2 mRNA and protein, that DAT is expressed in the plasma membrane and VMAT2 is expressed in cellular membranes, and suggest that macrophages may be able to take up extracellular dopamine and store it in vesicles.

### Activation of dopamine receptors modulates macrophage cytokine secretion

**Figure 5:**
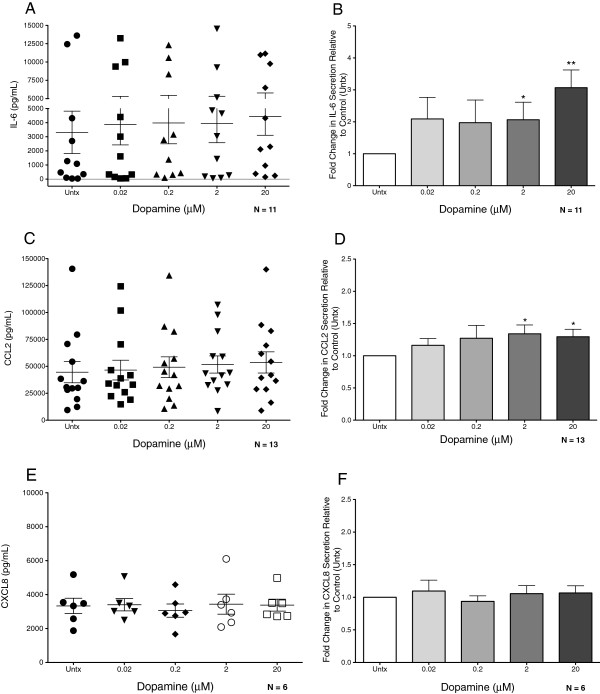
**Dopamine modulates macrophage cytokine secretion.** Monocyte-derived macrophages (MDM) were treated with different concentrations of dopamine (grey bars, 20 nM, 200 nM, 2 uM, and 20 uM) for 24 hrs. Control cells were treated with media (Untx, white bars). Supernatants were collected after treatment and analyzed for IL-6 (A, B, n = 11), CCL2, (C, D,n = 13) and CXCL8 (E, F, n = 6) by ELISA. The dot graphs (**A**, **C**, **E**) show the concentration of each cytokine in the culture medium after 24 hours for all donors examined, demonstrating the inter-donor variability in the secretion of each cytokine. Bar graphs (**B**, **D**, **F**) represent the mean of the dopamine–mediated fold change in cytokine from all donors relative to the control (Untx), which is set to 1. Significance was determined using a Wilcoxon Signed Rank Test (B, D) or two-tailed Student’s T-test (H). * P >0.05,** P >0.01.

**Figure 6:**
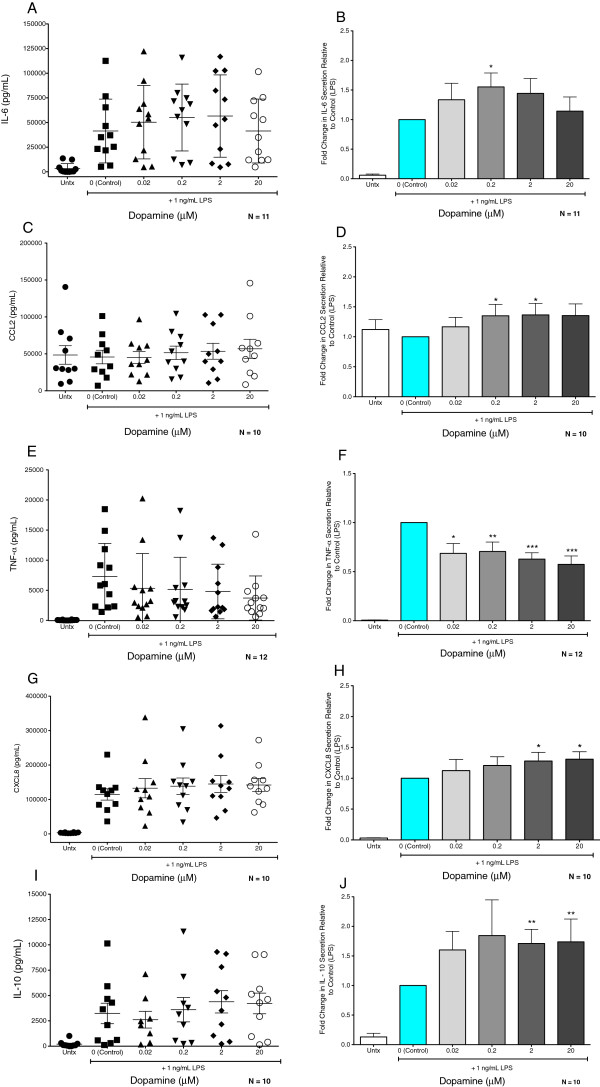
**Dopamine modulates LPS-induced macrophage cytokine secretion.** MDM were treated with different concentrations of dopamine (grey bars, 20 nM, 200 nM, 2 μM, and 20 μM) in conjunction with 1 ng/mL of LPS. Control cells (Control, blue bars) were treated with LPS only, while untreated MDM were treated with vehicle (Untx, white bars). Supernatants were collected after 24 hrs of treatment and analyzed for IL-6 (A, B, n = 11), CCL2 (C, D, n = 10), TNF-α (E, F, n = 12), CXCL8 (G, H, n = 10) and IL-10 (I, J, n = 10) by ELISA. The dot graphs (**A**, **C**, **E**, **G**, **I**) show the concentration of each cytokine in the culture medium after 24 hours for all donors examined, demonstrating the inter-donor variability in the secretion of each cytokine. Bar graphs (**B**, **D**, **F**, **H**, **I**) represent the mean of the dopamine mediated fold change in cytokines from all donors relative to the control (LPS only), which was set to 1. Significance was determined using a Wilcoxon Signed Rank Test (B, D) or two–tailed Student's t-test (H). * P >0.05, ** P >0.01, *** P >0.001.

To examine the effect of dopamine on cytokine secretion, MDM from different donors were treated with 20 nM, 200 nM, 2 μM or 20 μM dopamine for 24 hours in either media alone or in the presence of LPS (1 ng/mL). Control cells were treated with media alone (Untx) or LPS in the absence of dopamine. LPS was used to model the effect of dopamine on macrophage cytokine secretion in an inflammatory environment. Supernatants from each treatment condition were analyzed by ELISA for secretion of IL-6, CCL2, TNF-α, CXCL8 or IL-10. These cytokines were selected because of their important roles in the neuroinflammation and in the development of neurological diseases such as HAND and Alzheimer’s Disease. Cytokine secretion in control MDM was compared to cytokine secretion in cells treated with different concentrations of dopamine. It is important to note that the variability inherent in studying primary human cells resulted in large inter-donor differences in cytokine secretion. In MDM not treated with LPS, dopamine had the most pronounced effect on IL-6, significantly increasing its elaboration by 2 and 3 fold at 2 and 20 μM dopamine respectively (n = 11, Figure 5A, 5B). Dopamine also significantly increased macrophage secretion of CCL2 by approximately 1.3 fold at 2 μM and 20 μM dopamine (n = 13, Figure 5C, 5D), but did not have any significant effect on secretion of CXCL8 (n = 6, Figure 5E, 5F). Changes in TNF-αor IL-10 secretion were not examined in non-LPS treated macrophages because secretion of these cytokines was undetectable in samples from many donors. In all MDM, LPS treatment induced a significant increase in the secretion of all cytokines except CCL2, as compared to MDM not treated with LPS. Dopamine significantly increased IL-6 secretion in LPS treated macrophages by 1.5 fold at 200 nM (n = 11, Figure 6A, 6B). Dopamine modulated CCL2 production in LPS treated macrophages similarly to non-LPS treated macrophages, significantly increasing CCL2 production approximately 1.4 fold at 200 nM and 2 μM dopamine, although not at 20 μM dopamine (n = 10, Figure 6C, 6D). The only cytokine decreased by dopamine treatment was TNF-α, as dopamine significantly reduced secretion of this cytokine approximately −1.3 to −1.4 fold in LPS treated macrophages at all concentrations examined (n = 12, Figure 6E, 6F). Unlike in non-LPS treated cells, dopamine significantly increased CXCL8 in macrophages treated with LPS by approximately 1.3 fold at both 2 μM and 20 μM (n = 10, Figure 6G, 6H). Dopamine also significantly increased IL-10 secretion in LPS treated cells approximately 1.7 fold at 2 μM and 20 μM (n = 10, Figure 6I, 6J), and trended towards increases at lower dopamine concentrations, although these changes were not significant. These data demonstrate that dopamine regulates cytokine secretion in primary human macrophages.

## Discussion

Dopamine is an immunoregulatory molecule, modulating the functions of rodent macrophages as well as human T-cells, in addition to its role as a neurotransmitter. Its effects on human macrophages and microglia had not been well characterized. We hypothesized that in the CNS of drug abusers or others with increased extracellular dopamine, dopamine–mediated changes in macrophage or microglial function would alter the development of neuroinflammation and the progression of several neurological diseases. We therefore determined whether human primary MDM express dopamine receptors and the dopaminergic machinery to respond to, synthesize, and take up dopamine. We showed that MDM express mRNA for all DR, as well as for DAT, VMAT2, TH and AADC. We demonstrated that MDM express D3R and D4R as well as DAT on the cell surface, VMAT2 on cellular membranes, and TH and AADC. We also showed that dopamine modulates the secretion of cytokines in both untreated and LPS-treated MDM. These findings indicate that macrophages express the proteins needed to respond to dopamine and that dopamine modulates macrophage functions. 

Macrophages in the CNS in close proximity to neurons [52] are likely to be exposed to dopamine during spillover from the releasing synapse into the extrasynaptic space [53,54]. Extracellular dopamine concentrations in the human brain are not well characterized, but in rats the basal levels are estimated to be between 10 and 30 nM [4,54] and can be increased to 250 nM with a single electrical stimulus [3]. In both rodent and primate models, cocaine and methamphetamine increase extracellular dopamine in different regions of the brain [1,2,55] and studies using cyclic voltammetry measured drug-induced dopamine flux to be as high as 500 nM - 5 μM [56-58]. To model the effects of dopamine on macrophages in the brains of drug abusers and others with increased CNS dopamine, the concentrations of dopamine used in this study range from 20 nM to 20 μM. We previously demonstrated that 20 μM dopamine increases HIV replication in human macrophages [49]. Many other studies showed that dopamine concentrations within this range modulate different functions in rodent macrophages and microglia, as well as in human T cells [17-24]. Dopamine mediates its effects primarily through activation of DR on the PM [30], although other dopamine mediated signaling pathways have been suggested by studies in rodent macrophages [22]. Dopamine receptor mRNA or protein was shown on rodent microglia [19] and human T-cells [20,40,59]. In T-cells, specific DR were shown to effect cytokine secretion [20], activation of regulatory T-cells [17] or β1 integrin function [21]. In human macrophages DR subtypes had not been fully characterized. Our study shows that primary human macrophages have mRNA for all five subtypes of DR and express D3R and D4R in the PM. Our previous work showed MDM express D1R and D2R on the cell surface and showed specific activation of D2-like DR induces MAP kinase signaling [49]. Another study showed D1R in human macrophages [60], although its localization was not characterized. These studies demonstrate that DR are expressed on the surface of human macrophages and suggest that distinct DR subtypes may differentially modulate macrophage functions. 

The effects of dopamine can also be regulated by DAT, VMAT2, TH and AADC. Our data show that primary human MDM express these proteins,. The dopamine transporter is localized to the PM and VMAT2 is in cellular membranes. Whether these proteins have a role in macrophage function is still unclear, as DAT was not found in a rodent macrophage cell line [61], while VMAT2 was expressed in promyelocytic human U937 cells but not in monocytic THP-1 cells [46,48]. However, functional DAT is expressed in human T-cells [62-64]. Tyrosine hydroxylase was detected in rodent macrophages [65], human T-cells [17] and human macrophages from arthritic tissue, although not in macrophages from non-arthritic controls [48], while AADC was found in human U937 cells and human T-cells [38,47]. Dopamine production has not been characterized in human macrophages, but low amounts of dopamine are synthesized in rodent macrophages [65,66] and human PBMC [17,40,67], and AADC in U937 cells is enzymatically active [47]. Dopamine uptake in human macrophages has also not been studied, but dopamine has been detected in cytoplasmic vesicles of human U937 cells and in rodent macrophages [66,67]. Together with our data, these studies suggest that human macrophages may take up, store, and synthesize dopamine, but the functional consequences of these actions remain unclear. In human CD4+/CD25+ regulatory T-cells, dopamine release and reuptake regulates an autocrine/paracrine loop through activation of D1-like DR to regulate IL-10 and TGF-β production [17]. 

Our data show that dopamine also regulates IL-10 production in MDM, suggesting dopamine synthesis and/or dopamine uptake, storage and release may participate in regulation of macrophage cytokine production. Our data demonstrate that dopamine treatment alters macrophage production of IL-6, CCL2, TNF-α, CXCL8 and IL-10. We found that 2 and 20 μM dopamine significantly modulated secretion of all cytokines examined, while lower concentrations (20 and 200 nM) only altered TNF-α, IL-6 and CCL2 production by LPS-treated MDM. Lower concentrations of dopamine also showed a trend towards modulation of both IL-6 in untreated MDM and IL-10 in LPS treated MDM, but these effects were not significant. Dopaminergic modulation of cytokines has been shown in both rodent macrophages [18,22] and human T-cells [20,68] using similar concentrations of dopamine or using DR agonists. One study showed significant decreases in IL-2, IL-4 and IFN-γ production from anti-CD3 activated human T-cells at lower dopamine levels (5–25 nM) [43]. The trend towards higher concentrations of dopamine being needed to induce significant cytokine secretion suggests that dopamine concentrations must be elevated above physiological levels to alter macrophage cytokine secretion.

Treatment of macrophages with LPS was used to model inflammatory conditions within the CNS. The effects of dopamine on LPS-treated macrophages indicate that macrophages in an inflammatory environment may respond differently to dopamine than do macrophages in homeostatic conditions. Elevated levels of LPS during HIV infection [69], and in individuals with HIV-associated dementia [70], suggest that macrophages in people with NeuroAIDS may respond differently to dopamine due to LPS-mediated activation. In our study, CXCL8 was signifcantly increased by dopamine treatment of LPS-treated MDM but was unaffected by dopamine in untreated cells. In contrast, treatment with 2 and 20 μM dopamine increased IL-6 2-to-3 fold in untreated cells, but increased IL-6 only 1.5 fold, and only at 200 nM dopamine, in LPS-treated macrophages. These data suggest that LPS activation of MDM primes the cells to respond differently to dopamine than non-LPS treated cells. Further investigation of changes in dopamine–mediated modulation of macrophage functions in response to inflammatory stimuli is an area of ongoing research. Dopamine mediated changes in cytokines have important implications for neuroinflammation. Elevated levels of CCL2 and CXCL8 may result in recruitment of peripheral blood monocytes, T-cells and neutrophils [71,72], increased blood brain barrier permeability [72,73] and altered migration of CNS cells [74]. Increases in IL-6, CCL2 and CXCL8 are associated with activation of perivascular macrophages and glia, and with the development of neurological diseases such as HAND, Alzheimer’s disease, Parkinsons’s or Huntington’s disease, and multiple sclerosis [26,75-78].

Changes in cytokine production may also be beneficial, as decreases in TNF-α and increases in both IL-10 and CCL2 could limit neuroinflammation or be neuroprotective, depending on the kinetics and localization of their expression. For example, in Parkinson’s disease, in which TNF-α plays a central role in the loss of dopaminergic neurons [79], decreases in this cytokine could reduce neuronal toxicity and slow disease progression [80]. Increases in CCL2 inhibit HIV-Tat induced apoptosis in neurons and astrocytes [81] and increased IL-10 facilitates neuronal recovery after traumatic brain injury by reducing inflammatory cytokine production [82]. Thus, changes in CNS dopamine which modulate macrophage secretion of the cytokines examined in this study may alter the CNS response to injury and infection, and significantly affect the pathogenesis of several neurological diseases [25,75,83].

## Conclusion

Macrophages are central to CNS homeostasis, immune and inflammatory response. Thus, the effects of elevated dopamine on macrophages may have a profound impact on neuroinflammation and the CNS response to insult as well as on the development of neurological diseases including Alzheimer’s disease and HAND. The use of illegal drugs such as cocaine and methamphetamine increases CNS dopamine, and could therefore alter the development of neuroinflammation and modulate macrophage functions. Several therapeutic drugs also increase extracellular dopamine in the CNS, and may therefore have broader effects than treatment of a specific disease or condition. More detailed analyses of the actions of dopamine on macrophages are important for our understanding of the effects of legal and illegal dopaminergic drugs on the CNS. These studies will address the efficacy and safety of long-term treatment regimens as well as facilitate the development of novel interventional strategies for neurologic diseases in the context of drug abuse.

## Abbreviations

AADC: aromatic amino acid decarboxylase; BSA: bovine serum albumin; CNS: Central nervous system; DA: dopamine hydrochloride; DAPI: 4',6-diamidino-2-phenylindole; DAT: dopamine transporter; DR: dopamine receptor; D1R: dopamine receptor 1; ELISA: enzyme-linked immunosorbent assay; FITC: fluorescein isothiocyanate; HAND: HIV-associated neurocognitive disorders; HEPES: hydroxyethyl piperazineethanesulfonic acid; LPS: lipopolysaccharide; M-CSF: macrophage colony stimulating factor; MDM: Monocyte-derived macrophage; PBMC: peripheral blood mononuclear cells; PM: plasma membrane; PVDF: polyvinylidene fluoride; TBS: Tris-buffered saline; TH: Tyrosine hydroxylase; TNF: Tumor necrosis factor; VMAT2: vesicular monoamine transporter 2.

## Competing interests

The authors declare that they have no competing interests.

## Authors’ contributions

PJG designed the study with JWB, performed the immunoassays, subcellular fractionation and immunoblotting, and participated in the qRT-PCR with LC and the confocal microscopy with EAE, and drafted the manuscript. LC participated in the qRT-PCR with PJG and helped edit the manuscript. EAE performed the confocal microscopy with PJG and helped edit the manuscript. JWB participated in the design of the experiments with PJG, coordinated and helped to draft and edit the manuscript. All authors have read and approved the final version of this manuscript.
